# Longitudinal changes in sleep and sleep-related symptoms among Korean adults between 2010 to 2022, including the COVID-19 pandemic period

**DOI:** 10.1371/journal.pone.0311600

**Published:** 2024-11-07

**Authors:** Hea Ree Park, Seo-Young Lee, Hye-Jin Moon, Jee Hyun Kim, Jae Wook Cho, Yong Won Cho, Chang-Ho Yun, Su-Hyun Han, Min Kyung Chu

**Affiliations:** 1 Department of Neurology, Inje University College of Medicine, Ilsan Paik Hospital, Goyang, Republic of Korea; 2 Department of Neurology, College of Medicine, Kangwon National University, Chuncheon, Republic of Korea; 3 Interdisciplinary Graduate Program in Medical Bigdata Convergence, Kangwon National University, Chuncheon, Republic of Korea; 4 Department of Neurology, Soonchunhyang University Bucheon Hospital, Bucheon, Republic of Korea; 5 Department of Neurology, Ewha Womans University Seoul Hospital, Ewha Womans University College of Medicine, Seoul, Republic of Korea; 6 Department of Neurology, Pusan National University Yangsan Hospital, Pusan National University School of Medicine, Yangsan, Republic of Korea; 7 Department of Neurology, Keimyung University School of Medicine, Daegu, Republic of Korea; 8 Department of Neurology, Seoul National University Bundang Hospital and Seoul National University College of Medicine, Seongnam, Republic of Korea; 9 Department of Neurology, Chung-Ang University Hospital, Chung-Ang University College of Medicine, Seoul, Republic of Korea; 10 Department of Medicine, Graduate School Kangwon National University, Chuncheon, Republic of Korea; 11 Department of Neurology, Severance Hospital, Yonsei University College of Medicine, Seoul, Republic of Korea; Arba Minch University, ETHIOPIA

## Abstract

**Background and purpose:**

The coronavirus disease 2019 (COVID-19) pandemic has significantly impacted people’s lifestyles, changing sleep patterns. This study investigated changes in sleep patterns and disturbances in South Koreans over the past decade, including during the pandemic.

**Methods:**

We compared data from the Korean Sleep Headache Society Phase I survey (n = 2484; conducted in 2010) and the National Sleep Survey of South Korea 2022 (n = 3729; conducted in 2022), involving participants aged 20–69 years. Changes in sleep schedule, sleep duration, social jet lag, insomnia, and daytime sleepiness were explored.

**Results:**

Workday bedtimes were advanced and free-day bedtimes and workday and free-day waking times were delayed during the pandemic. Increased circadian preference for eveningness and social jet lag were noted. A significant decrease in sleep duration and sleep efficiency, along with an increased prevalence of insomnia and daytime sleepiness, was noted with age- and sex-specific variations.

**Conclusions:**

Over the past decade, including during the COVID-19 pandemic, sleep habits have changed significantly and sleep problems worsened. This study emphasize the need for more comprehensive public health strategies and research to facilitate sleep recovery in the post-pandemic period within a society known for its high prevalence of sleep deprivation.

## Introduction

South Korea has a high prevalence of sleep deprivation [1]. Given profound impact of sleep deprivation on health [2], the importance of sufficient sleep cannot be overemphasized. However, the worldwide lockdowns owing to the coronavirus disease 2019 (COVID-19) pandemic have negatively impacted the psychological well-being of people, with a significant impact on sleep. Previous studies have already examined changes in sleep during the global pandemic and reported decreased sleep quality and increased insomnia worldwide [3–7]. However, changes in sleep duration and patterns vary across studies. Some studies have reported phase delays and decreased sleep duration [4], while others have reported increased sleep duration owing to reduced social commitment during the pandemic [6, 8]. These inconsistent results may be attributed to several factors, including participants’ pre-pandemic routine lifestyles, forms and intensities of social restrictions during the pandemic, and cultural variations between countries.

South Korea implemented strict measures to prevent the disease spread during the pandemic [9]. Therefore, it is reasonable to suspect that such significant changes in daily life have considerably impacted the sleep patterns of South Koreans. However, unlike other countries, South Korea lacks research on the changes in sleep during the pandemic. Although a recent study reported a high prevalence of insomnia during the pandemic in Korea [10], it did not investigate changes in sleep patterns from the pre-pandemic period.

It’s important to understand the potential compounding effects of the pandemic, given the existing sleep issues in South Korea. Therefore, we investigated the changes in sleep and sleep-related discomfort over more than a decade, including the COVID-19 pandemic period, by examining nationally-representative sleep survey data.

## Materials and methods

### Participants and survey procedure

**Fig 1:**
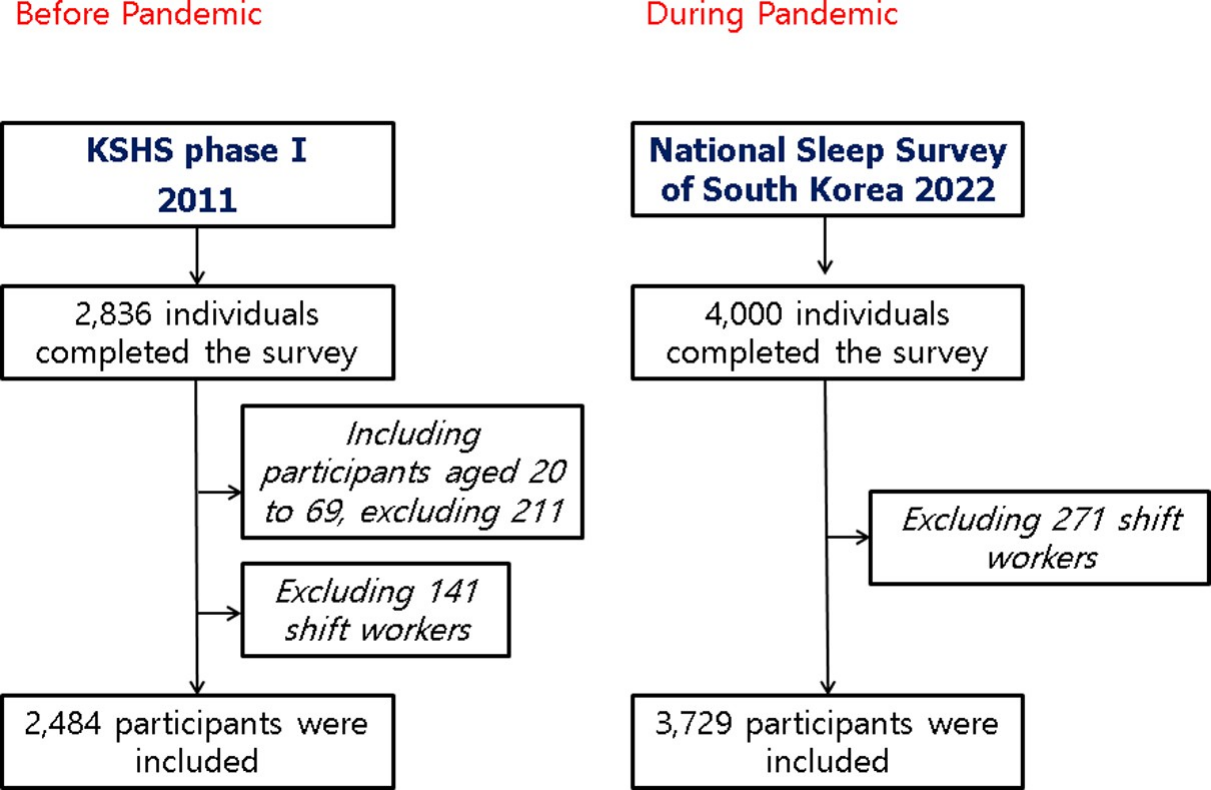
Study flow chart. KSHS, Korean Sleep Headache Society.

This study used two nationally representative sleep survey data in South Korea; the Korean Sleep Headache Society (KSHS) Phase I survey and the National Sleep Survey of South Korea 2022 (NSSK). Fig 1 outlines the study flowchart.

The KSHS Phase I survey is a nationwide population-based survey, conducted by Gallup Korea in 2010, using face-to-face interviews, and used a multistage clustered sampling from 15 administrative districts (except Jeju Island) [11]. The researchers approached 7,615 individuals according to the population distributions of sex and age group and obtained responses from 2,836. Of them, we excluded 211 respondents aged < 19 or ≥ 70 years for consistency with the NSSK age range. We also excluded 141 night or shift workers who exhibited sleep habits markedly different from those of the general population. Thus, 2,484 participants from the KSHS Phase I survey were included in this study.

The NSSK is an online survey of adults aged 20–69 in South Korea, using a structured questionnaire [12]. This survey was commissioned by the Epidemiology Committee of the Korean Sleep Research Society and conducted by conducted by Embrain Public Company from January to February 2022. This period coincided with the start of the fifth wave of the COVID-19 pandemic (January to June 2022), when government-led robust social distancing efforts were implanted. A nationally representative sample of 4,000 participants was constructed using stratified multistage random sampling according to age, sex and residence. To ensure consistency with the characteristics of the KSHS survey participants, 271 night or shift workers were excluded from the NSSK survey, resulting in a sample size of 3,729 participants.

### Sociodemographic characteristics

We collected demographic factors including age, sex, educational level, income, and current working status. The level of education was categorized as follows based on years of education: middle school or less (≤9 years), high school (9–12 years), or university/college or more (≥12 years). Monthly income level was divided into three groups: <2 million KRW (minimum monthly cost of living per person), 2–5 million KRW, and >5 million KRW. Alcohol consumption was classified according to drinking frequency (never, less than once a week, once or twice a week, 3–4 times per week, and 5 or more times per week), while smoking status was classified as never, former, or current smoker.

### Sleep pattern measures

We investigated the participants’ typical bedtime (hh:mm), wake time (hh:mm), and sleep duration (hh:mm) on workdays and free days. Time in bed (TIB) was calculated using wake time and bedtime for both workdays and free days, and average TIB was computed using the formula: (workday TIB × 5 + free-day TIB × 2)/7. Sleep duration on workdays and free days was obtained through the question: "On average, how many hours did you sleep during the past month?" The average sleep duration was calculated as follows: (workday sleep duration × 5 + free-day sleep duration × 2)/7. Sleep efficiency was estimated as the ratio of average sleep duration to average TIB. To quantify social jet lag (SJL), we calculated midsleep point on free days (MSF) and midsleep point on workdays (MSW) using the following formula: SJL = |MSF ‒ MSW|. The definition and measurements were based on the theory of Wittmann et al [13]. And we employed MSF corrected for work day sleep debt (MSFsc) to measure chronotype, using the formula: MSFsc = MSF ‒ (free-days sleep duration ‒ average sleep duration)/2 [14]. Higher MSFsc values indicate later chronotype.

### Insomnia and daytime sleepiness

To investigate insomnia symptoms, we utilized the Korean version of the Insomnia Severity Index (ISI-K) [15]. The ISI-K consists of seven items that assess difficulty falling asleep, difficulty staying asleep, the problem of waking up too early, satisfaction with current sleep patterns, interference with daily functioning, noticeable impairment from sleep disturbances, and distress from sleep problems. The total score ranges from 0 to 28, with higher scores indicating a greater severity of insomnia. Participants with an ISI-K score of ≥15 were classified as having moderate-to-severe insomnia. In this study, we evaluated the frequency of moderate-to-severe insomnia using difficulty falling asleep (ISI-1a), difficulty staying asleep (ISI-1b), and waking up too early(ISI-1c) (i.e., ISI-1a, b, c ≥ 2). We also investigated the current use of sleeping pills and other medications.

The assess excessive daytime sleepiness (EDS), we used Epworth Sleepiness Scale (ESS), with a score or 11 or more defined as EDS.

### Statistical analysis

We compared participants’ sociodemographic characteristics, sleep pattern measures, insomnia, and daytime sleepiness between the surveys in 2010 and 2022 using chi-square and Student’s t-tests. Considering the significant differences in age, income level, education level, alcohol consumption, smoking status, and current working status between the two surveys, we adjusted for these variables. Binomial logistic regressions for categorical variables and linear regressionss for continuous variables, with adjustments for age, income, education, alcohol consumption, smoking, and working status, were used to compare sleep data between the two surveys. This adjustment method is consistent with the approach used in previous studies comparing two surveys with different sociodemographic characteristics [16]. In addition, subgroup analyses according to age (20–29, 30–39, 40–49, 50–59, and 60–69 years) and sex were conducted to analyze sleep schedules and insomnia. SPSS Statistics (version 25; IBM Corp., Armonk, NY, USA) was used for statistical analyses. The criterion for statistical significance was set at p<0.05.

## Results

### Sociodemographic characteristics

Table 1 summarizes the sociodemographics of the participants in the KSHS Phase I (2010, pre-pandemic) and NSSK 2022 (2022, intra-pandemic). The NSSK 2022 survey’s participants were slightly older than KSHS Phase I’s participants (mean age, 44.7±13.3 vs. 43.5±13.4 years; p<0.001), while sex ratios in the two surveys were similar. Education level, income level, and employment status improved, whereas frequent alcohol consumption (≥3/week) and the number of participants currently smoking decreased in 2022. BMI did not differ between the two surveys.

**Table 1:** Sociodemographic characteristics.

Variables	Group	2010 (N = 2484)	2022 (N = 3729)	*p*
Sex (%)	Male	1210 (48.7)	1847 (49.5)	0.544
	Female	1274 (51.3)	1882 (50.5)	
Age		43.5 ± 13.3	44.7 ± 13.3	<0.001
Age group (%)	20–29	445(17.9)	679 (18.2)	<0.001
	30–39	577 (23.2)	672 (18.0)	
	40–49	584 (23.5)	815 (21.9)	
	50–59	504 (20.3)	847 (22.7)	
	60–69	374 (15.1)	716 (19.2)	
Body mass index		23.7 ± 15.5	23.7 ± 3.7	0.923
The level of Education (%)	Middle school or less	367 (14.9)	32 (0.9)	<0.001
	High school	1122 (45.6)	848 (22.7)	
	College or more	971 (39.5)	2849 (76.4)	
Monthly income level (%)^*^	< 2,000,000	362 (14.6)	494 (13.2)	<0.001
	2,000,000–4,999,999	1681 (67.7)	1819 (48.8)	
	≥ 5,000,000	441 (17.8)	1416 (38.0)	
Alcohol (%)	Never	858 (34.5)	1228 (32.9)	<0.001
	Less than once a week	725 (29.2)	1381 (37.0)	
	Once or twice a week	327 (13.2)	741 (19.9)	
	3–4 times week	482 (19.4)	276 (7.4)	
	5 or more times/week	92 (3.7)	103 (2.8)	
Smoking (%)^*^	Current smoker	677 (27.4)	734 (19.7)	<0.001
	Ex-smoker	286 (11.6)	969 (26.0)	
	Never	1510 (61.1)	2026 (54.3)	
Current working status (%)	Emplyed	1617 (65.1)	2581 (69.2)	0.001
	Unemployed	867 (34.9)	1148 (30.8)	

*Some values are missing owing to a lack of responses.

### Changes in sleep habits, insomnia, and EDS

**Fig 2:**
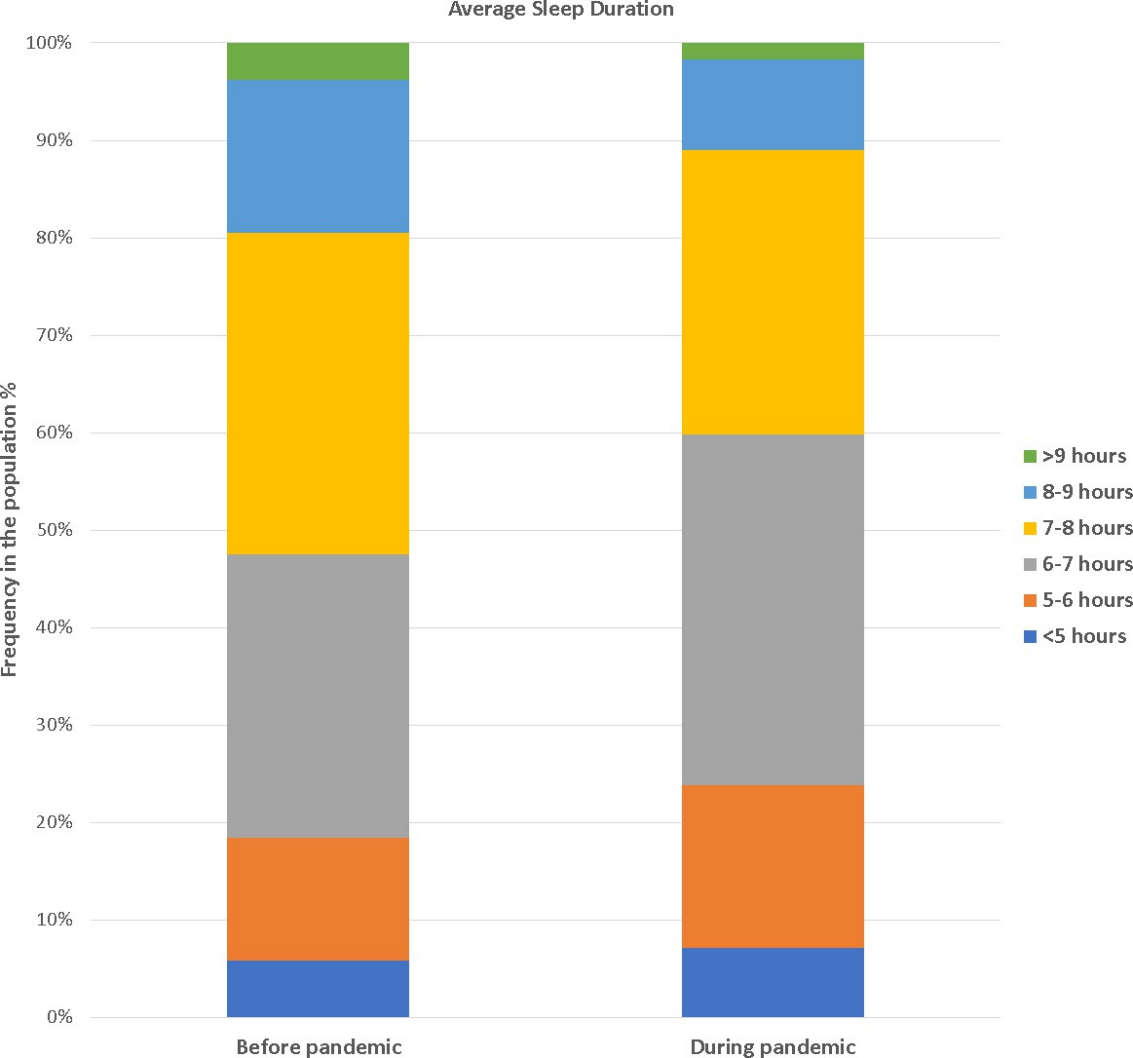
Changes in the distribution of average sleep duration.

Table 2 presents the differences in sleep habits and sleep-related questionnaire answers between the two surveys. The mean bedtime was advanced by 2 min on workdays and delayed by 20 min on free days during the pandemic. Moreover, the mean wake-up time during the pandemic was delayed by 23 min on workdays and by 53 min on free days. The mean TIB increased by 22 min on workdays and by 28 min on free days. Meanwhile, the average sleep duration was reduced by 25 min, from 7.20 h to 6.78 h. Specifically, the proportion of short sleepers (<6 h) increased from 18.4% to 23.8%, while that of long sleepers (≥8 h) decreased from 19.5% to 11.0% (Fig 2). Sleep efficiency reduced from 98.3% to 88.2%. The mean MSFsc during the pandemic was delayed by 30 min, from 03:34 to 04:04, while the mean SJL increased by 28 min, from 37 min to 65 min. These changes remained significant after adjusting for age, education, monthly income, alcohol consumption, smoking status, and employment status (Table 3).

**Table 2:** Changes in sleep habits and sleep-related symptoms.

Variables	2010 (N = 2484)	2022 (N = 3729)	*p*
Bedtime_workdays, clock time	23:44 ± 1:27	23:43 ± 1:35	0.428
Bedtime_free days, clock time	23:56 ± 1:28	24:15 ± 1:46	<0.001
Wake-up time_workdays, clock time	6:53 ± 1:29	7:15 ± 1:38	<0.001
Wake-up time_free days, clock time	7:43 ± 1:54	8:36 ± 1:54	<0.001
Time in bed_workdays, hh:mm	7.17 ± 1.45	7.53 ± 1.37	<0.001
Time in bed_free days, hh:mm	7.81 ± 1.63	8.28 ± 1.60	<0.001
Average time in bed, hh:mm	7.35 ± 1.34	7.74 ± 1.30	<0.001
Social jet lag, min	36.86 ± 53.93	64.60 ± 66.59	<0.001
Sleep duration, h	7.20 ± 1.23	6.78 ± 1.14	<0.001
Sleep efficiency, %	98.32 ± 5.47	88.21 ± 10.78	<0.001
Chronotype, MSFsc, clock time	3:34 ± 1:54	4:5 ± 1:57	<0.001
Insomnia severity index (ISI)	3.72 ± 4.62	9.33 ± 4.61	<0.001
Moderate-to-severe insomnia(ISI≥15), n(%)	100 (4.0)	471 (12.6)	<0.001
Difficulty initiating sleep (ISI1a≥2), n(%)	233 (9.4)	938 (25.2)	<0.001
Difficulty maintaining sleep (ISI1b≥2), n(%)	202 (8.1)	831 (22.3)	<0.001
Waking up too early (ISI1c≥2), n(%)	292 (11.8)	838 (22.5)	<0.001
Intake of sleeping pills, n(%)	58 (2.3)	66 (1.8)	0.142
Epworth sleepiness scale (ESS)	5.56 ± 3.93	6.48 ± 3.58	<0.001
Excessive daytime sleepiness (ESS>10), n(%)	285 (11.5)	478 (12.8)	0.123

MSF_SC_, midsleep point on free days corrected for sleep debt on workdays

**Table 3:** Regression analysis of sleep habits and sleep-related symptoms associated with pandemic status.

Variables	Crude ß / OR (95% CI)	Crude *p*	Adj. OR ß / OR ^*^ (95% CI)	Adj. p^*^
Bedtime_workdays	-0.03 (-0.11,0.05)	0.436	-0.01 (-0.1,0.07)	0.748
Bedtime_free days	0.32 (0.24,0.41)	< 0.001	0.31 (0.22,0.4)	< 0.001
Wake-up time_workdays	0.38 (0.3,0.46)	< 0.001	0.45 (0.37,0.54)	< 0.001
Wake-up time_free days	0.88 (0.78,0.98)	< 0.001	0.91 (0.82,1.01)	< 0.001
Time in bed_workdays	0.36 (0.29,0.43)	< 0.001	0.43 (0.35,0.51)	< 0.001
Time in bed_free days	0.47 (0.39,0.55)	< 0.001	0.52 (0.42,0.61)	< 0.001
Average time in bed	0.39 (0.33,0.46)	< 0.001	0.46 (0.38,0.53)	< 0.001
Social jet lag	27.74 (24.6,30.88)	< 0.001	27.38 (23.85,30.91)	< 0.001
Sleep duration	-0.31 (-0.36, -0.27)	< 0.001	-0.31 (-0.36, -0.26)	< 0.001
Sleep efficiency	-0.27 (-0.28, -0.25)	< 0.001	-0.25 (-0.27, -0.23)	< 0.001
Chronotype, MSFsc	0.51 (0.41,0.61)	< 0.001	0.55 (0.44,0.66)	< 0.001
Insomnia severity index (ISI)	5.62 (5.38,5.85)	< 0.001	5.88 (5.61,6.15)	< 0.001
Moderate-to-severe insomnia(ISI≥15)	3.45 (2.76,4.3)	< 0.001	4.11 (3.18,5.32)	< 0.001
Difficulty initiating sleep (ISI1a≥2)	3.25 (2.78,3.79)	< 0.001	4.22 (3.51,5.06)	< 0.001
Difficulty maintaining sleep (ISI1b≥2)	3.24 (2.75,3.81)	< 0.001	3.69 (3.04,4.48)	< 0.001
Waking up too early (ISI1c≥2)	2.18 (1.88,2.51)	< 0.001	2.71 (2.27,3.24)	< 0.001
Intake of sleeping pills	0.75 (0.53,1.08)	0.120	0.86 (0.56,1.33)	0.508
Epworth sleepiness scale (ESS)	0.92 (0.73,1.11)	< 0.001	1.1 (0.88,1.32)	< 0.001
Excessive daytime sleepiness (ESS>10), n(%)	1.13 (0.97,1.33)	0.114	1.29 (1.07,1.55)	0.006

ß coefficients are presented for continuous variabls, and OR are presented for categorical variables.

*Adjusted for age, education, monthly income level, alcohol consumption, smoking status, and employment status.

OR, odds ratios; CI, confidence interval; MSF_SC_, midsleep point on free days corrected for sleep debt on workdays.

The mean ISI score was significantly higher during the pandemic than before it (9.3±4.6 vs. 3.7±4.6, p<0.001), and the prevalence of moderate-to-severe insomnia (ISI≥15) also increased during the pandemic, from 4.0% to 12.6%. All insomnia symptoms (difficulty in sleeping, frequent arousal, and waking too early) were more frequent during the pandemic. However, no significant difference was noted in the proportion of participants taking hypnotics.

The mean ESS score was significantly higher during the pandemic than before it (6.5±3.6 vs. 5.6±3.9, p<0.001). The prevalence of EDS (ESS>10) was not significantly different (12.8% vs. 11.5%, p = 0.114) in the unadjusted comparison; however, it was significantly higher during the pandemic in logistic regression analyses after adjusting for age, education, income, alcohol consumption, smoking status, and working status (Adjusted odds ratio 1.29 [95% confidence interval 1.07–1.55], p = 0.006).

### Age- and sex-specific differences in changes in sleep habits, insomnia, and EDS

**Fig 3:**
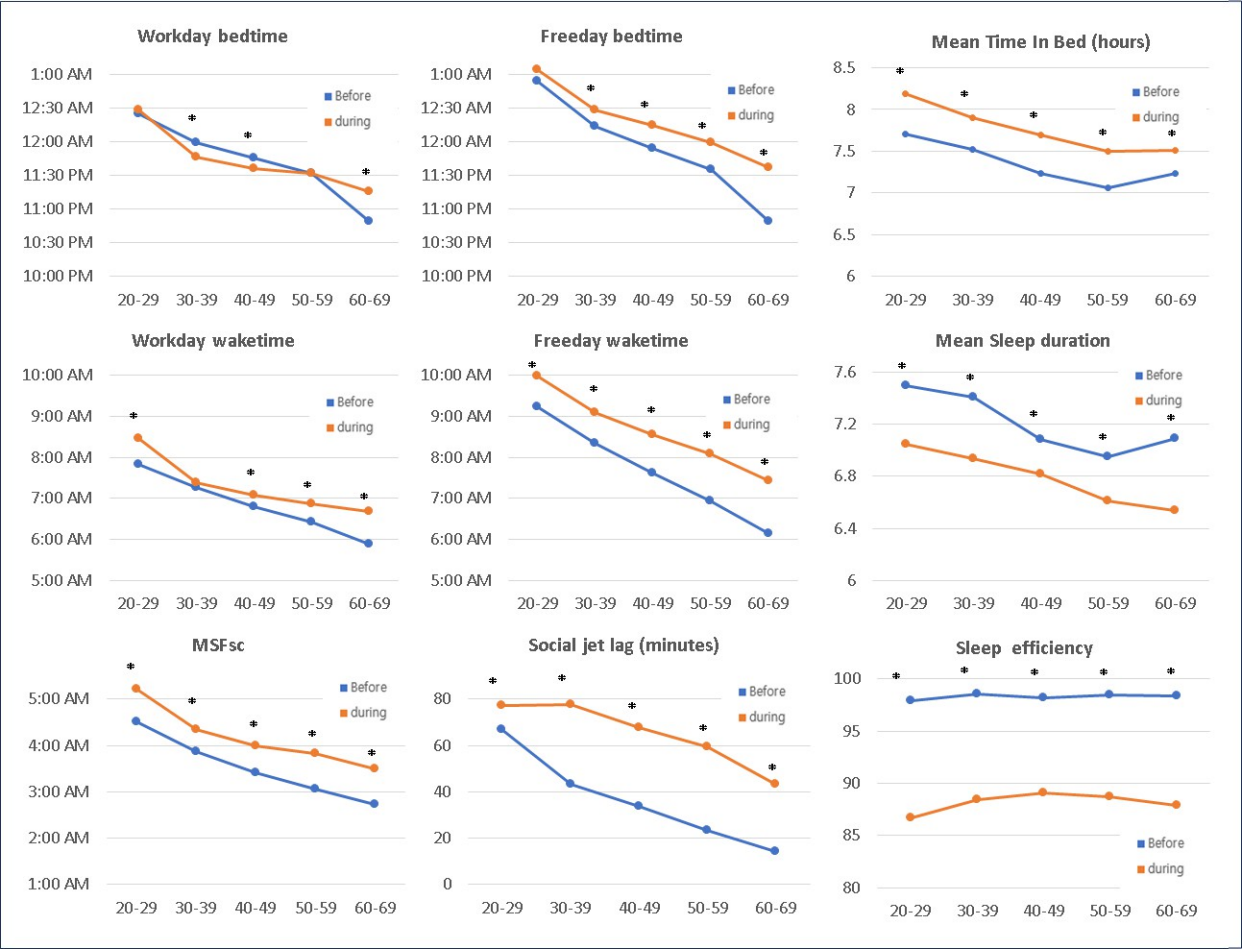
Changes in sleep patterns before versus during the COVID-19 pandemic by age. MSFsc, midsleep point on free days corrected for sleep debt on workdays.

Fig 3 presents the distinct patterns of changes in sleep habits before and during the pandemic according to age. While bedtime on workdays was slightly advanced in participants aged 30–50 years, it was significantly delayed in those aged 60–69 years. A delay in bedtime on free days was observed across all age groups, except in those aged 20–29 years, and was more profound in those aged 60–69 years. Wake times on workdays and free days were also delayed in all age groups (except for workday wake times in those aged 30–39 years) but were more prominent in those aged 50–59 and 60–69 years. The delay in MSFsc was greater in the 50–59- and 60–69-year age groups than in the 30–39-year age group, indicating a trend of reduced age-related differences in MSFsc. The SJL increased in all age groups. An increase in TIB was observed in all age groups and was most prominent in the 20–29-year group. However, mean sleep duration decreased significantly in all age groups. Consequently, sleep efficiency decreased across all age groups, with the most notable decrease in the 20–29-year group.

**Fig 4:**
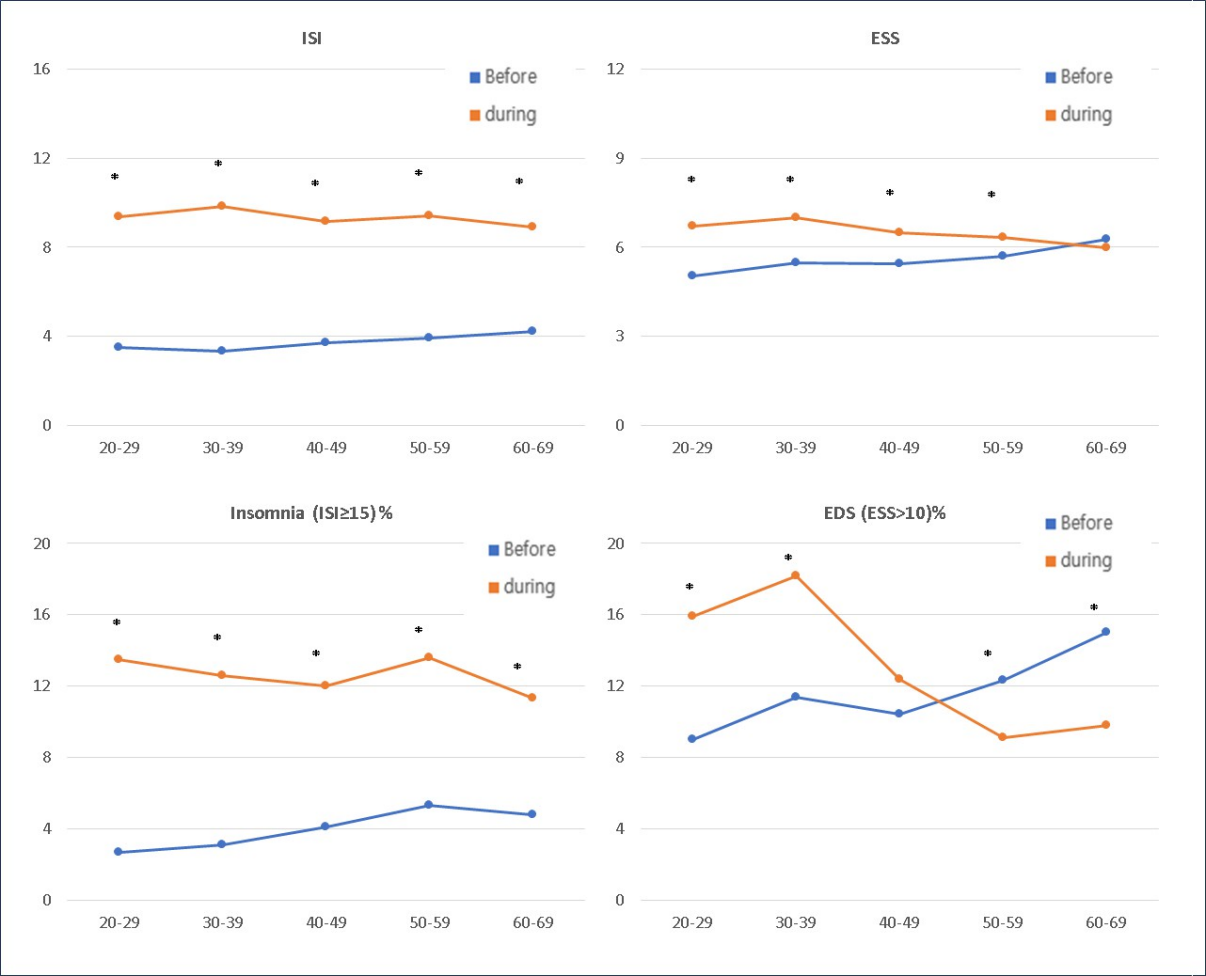
Changes in symptoms of insomnia and excessive daytime sleepiness during the COVID-19 pandemic by age. ISI, Insomnia Severity Index; ESS, Epworth Sleepiness Scale; EDS, excessive daytime sleepiness (ESS score >10).

Fig 4 shows the age-related changes in insomnia and EDS according to age. Mean ISI and prevalence of insomnia (ISI≥15) increased across all age groups but was more prominent in the 20–29- and 30–39-year groups. The mean ESS increased in all age groups, except in the 60–69-year group, and was more notable in the 20–29- and 30–39-year groups. The prevalence of EDS (ESS>10) increased in the 20–29 and 30–39 age groups but decreased in the 50–59 and 60–69 age groups.

Sex differences were less pronounced than age differences among participants. An increase in bedtime on workdays was observed in men; however, no significant changes were observed in women. Delays in bedtime on free days and wake-up time on both workdays and free days were more prominent in women. The increase in mean TIB was greater in women, but sleep duration decreased in both sexes, leading to a more pronounced decrease in sleep efficiency in women than in men. The mean ISI score and prevalence of insomnia increased similarly in both men and women. In contrast, the prevalence of EDS increased only in men. Detailed information on the sex-specific patterns of change is provided in S1 Table.

## Discussion

This is the first report on longitudinal changes in sleep habits and disturbances in Korean adults population over more than a decade, including the COVID-19 pandemic period. This study also demonstrated age- and sex-specific differences in sleep habits and sleep disturbance trends. From 2010 to 2022, bedtimes on workdays were slightly advanced, whereas those on free days and wake-up times on workdays and free days were significantly delayed. This shift resulted in a trend of eveningness of the circadian preference and increased SJL. Although the mean TIB increased, the average sleep duration decreased, with a higher prevalence of short sleepers (<6 h). Sleep efficiency declined, and the prevalence of insomnia and EDS increased.

The observed changes in sleep patterns were multifaceted and related to shifts in daily routines. The slight advancement in workday bedtime may be attributed to the expansion of remote work (working from home) and a decline in evening social engagement, such as after-work gatherings, during the pandemic period. This was supported by the results of subgroup analysis revealing that this change was more pronounced in the 30–50-year groups and in men. Our findings of advanced bedtimes on workdays during the pandemic differed from previous studies in other countries, which found delayed bedtimes or mixed results [4, 6, 17–19]. This difference may stem from the differences in pre-pandemic baseline lifestyles between countries, such as working hours and home return times. Long working hours have been reported in South Korea, suggesting that greater changes in lifestyle patterns contribute to distinct sleep pattern changes [20].

In contrast to the marginally advanced mean bedtime on workdays, we found a significant delay in bedtime on free days and waking time on both workdays and free days. Consequently, there was a notable delay in MSFsc, indicating a shift in the chronotype toward eveningness. The delay in the waking time on workdays may be linked to the elimination of commute times owing to remote work or online schooling. However, this factor alone cannot explain delays in bedtime and wakeing times on free days. A more plausible explanation is that there has been a chronotype shift toward evening in Korean adults, over the decade, including the pandemic.

Physical activity and light exposure are commonly associated with circadian robustness [21]. Exposure to electronic media devices has increased dramatically in modern society. The light from electronics can suppress the production of melatonin, a sleep-promoting hormone, and delay circadian rhythms [22, 23]. Increases in screen time during pandemic period have also been reported in multiple countries [24–26]. Reduced physical activity and decreased exposure to natural light during the daytime, combined with an increase in light exposure at night due to increased screen time, have been related to circadian phase delay during first waves of lockdown [27]. Sleep pattern changes and delayed circadian rhythm observed in this study might have been influenced by adverse changes in lifestyle that include reduced physical activity and negative changes in light during the pandemic.

Social restrictions during the pandemic period have impacted work–life schedules, increasing the prevalence of remote work [28, 29]. With more flexible schedules and autonomy, the severity of SJL has improved in several countries [30–32]. However, we also observed a paradoxical increase in SJL. One explanation for this could be the variation in the level of remote work by country during the pandemic. The proportion of remote workers to total employees was only 5.4% in 2021 and 4.4% in 2022 in South Korea [33, 34], compared to 40.1% and 27.5%, respectively, in the United States [35]. In Europe, nearly half (48%) of all employees worked from home for at least some time during the COVID-19 pandemic and more than a third (34%) worked exclusively from home [28]. Although obtaining directly comparable data from different countries is challenging, working from home may be less common in developed Asian countries, including South Korea, because of factors such as the ability to control disease outbreaks and house size [36]. Therefore, although the proportion of teleworking or flexible work in South Korea increased during the pandemic, it may not have been sufficient to improve SJL. Instead, a significant delay in chronotype over a decade, including during the pandemic period, may have contributed to the increase in SJL in the South Korean population.

In this study, the delay in sleep timing was greater in middle-aged and older adults, with a consequent convergence of sleep patterns across age groups. This trend may be related to the widespread use of smartphones and online video platforms such as YouTube across all ages in South Korea. The penetration rate of smartphones in Koreans aged ≥55 years was 69.1% in 2018 and has increased rapidly in recent years [37]. In addition, the trend toward early retirement may have related to these changes [38]. Retirement is typically related to increased sleep duration and later bedtime and waking time [39].

Despite an increase in TIB during the COVID-19 pandemic resulting from changes in sleep habits, there was a noticeable decrease in sleep duration. Factors contributing to this change include psychosocial distress, reduced outdoor activity, increased screen time, and disruption of routine schedules such as irregular working hours owing to remote work. This could also explain the pronounced increase in the prevalence of insomnia observed in our study, in which all types of insomnia complaints increased. These trends are consistent with those of global studies reporting deteriorated sleep quality and increased prevalence of insomnia, along with increased consumption of sleeping pills during the pandemic [3–7, 19].

Another notable aspect was the variation in changes in insomnia and EDS across age groups and sexes. Increased insomnia was observed across all age groups but was particularly prominent in the 20–29 and 30–39 age groups. The pattern of EDS changes differed across all demographics, being significantly more prevalent only in the 20–29-year age group and in men. Young adults, especially men who typically engage in more active social and outdoor lifestyles, experience more significant changes. Several studies reported heterogeneity in sleep changes caused by the COVID-19 pandemic, and our study highlights that age and sex are crucial factors in determining the impact of the pandemic on sleep [4, 6, 17, 40].

The changes in sleep patterns and phase delay seen in this study are over a period of more than a decade. In Finland, increased self-reported definite eveningness and delayed mid-sleep points were found between 2007 and 2017, which was speculated to be due to temporal change of social and behavioral aspects [41]. In South Korea, delayed wake-up times were reported by two pre-pandemic, longitudinal studies; the KSHS survey (from 2009 to 2018) [42] and the Korean Time Use Survey (KTUS) (from 20014 to 2019) [43]. However, there was no significant change in MSFsc in the KSHS survey [42]. In our study, we included the pandemic period in analyses, and found significant circadian phase delays. The changes in sleep patterns observed in our findings may be the result of the unique impact of the pandemic, combined with social and behavioral changes over a decade or more in South Korean society, where long working hours and sleep deprivations are already prevalent.

This study had some limitations. First, the comparison of sleep patterns between the two national surveys might reflect a combination of changes attributable to the COVID-19 pandemic with those occurring over a decade ago. A longitudinal Korean study comparing data from 2010 to 2018 demonstrated various changes in sleep patterns [42]. A comparison with more recent pre-pandemic or post-pandemic data would provide a clearer picture of the changes attributable to the pandemic and accompanying social isolation. Second, relying solely on the participants’ subjective reports might have introduced a recall bias, as individuals tend to base their responses on recent days of experience or times rounded to the nearest half or full hour [44]. Third, the study did not examine whether the participants worked remotely. Assessing the proportion of remote workers would have better explained the reasons for the changes in SJL. Forth, we did not ascertain whether participants discontinued using alarm clocks on free days in either of the surveys, which could potentially introduce a bias in the calculation of chronotype and SJL [45]. Finally, there was a methodological difference in our study. The pre-pandemic KSHS survey data was collected in person, while the NSSK survey data during the COVID-19 pandemic was collected online. The shift from in-person to online surveys may introduce variations in response accuracy and bias. In particular, despite using the same questions, the significant differences in sleep duration and sleep efficiency between the two surveys may suggest a potential bias due to differences between online and offline survey.

In conclusion, this report provides the first, detailed analyses of longitudinal changes in sleep habits and disturbances over a 10-year period, including the COVID-19 pandemic period, in a South Korean adults. There were significant changes in sleep and chronotypes, and worsening sleep disturbances due to downward trends in sleep habits. As times have changed, people’s sleep patterns have changed, and the pandemic has also affected this, and this study highlights the need for sleep recovery after the pandemic. The study findings also highlighted significant age and sex differences in these changes. These insights underscore the need for continued research and comprehensive age- and sex-specific public health strategies to improve sleep, post-COVID-19 in Korean, where sleep deprivation is already prevalent.

## Supporting information

S1 Table Patterns of change in sleep habits, insomnia, and excessive daytime sleepiness according to sex. (DOCX)
