# Supplementary material for: Longitudinal changes in sleep and sleep-related symptoms among Korean adults between 2010 to 2022, including the COVID-19 pandemic period
Source: PLoS One. 2024 Nov 7;19(11):e0311600. doi: 10.1371/journal.pone.0311600 (PMC11542832; doi:10.1371/journal.pone.0311600)
Supplement: S1 Table — (DOCX) [file pone.0311600.s001.docx]

**S1 Table.** Patterns of change in sleep habits, insomnia, and excessive daytime sleepiness according to sex

| **Variables** | **Before pandemic** | |  | **During pandemic** | | |
| --- | --- | --- | --- | --- | --- | --- |
|  | (n= 2484) | |  | | (N=3729) | |
|  | Men  (n=1210) | Women |  | | Men  (n=1847) | Women |
| Bedtime_workdays, clock time | 23:50 | 23:38 |  | | 23:41^†^ | 23:43 |
| Bedtime_free days, clock time | 24:02 | 23:49 |  | | 0:13^†^ | 0:17^†^ |
| Wake-up time_workdays, clock time | 6:56 | 6:48 |  | | 7:05^†^ | 7:24^†^ |
| Wake-up time_free days, clock time | 7:47 | 7:38 |  | | 8:25^†^ | 8:45^†^ |
| Average Time in bed, h | 7.31 | 7.38 |  | | 7.60^†^ | 7.89^†^ |
| Social jet lag, min | 36.8 | 36.9 |  | | 63.8^†^ | 65.4^†^ |
| Sleep duration, h | 7.17 | 7.23 |  | | 6.70^†^ | 6.86^†^ |
| Sleep efficiency, % | 98.5 | 98.2 |  | | 88.9^†^ | 87.6^†^ |
| Chronotype, MSFsc, clock time | 3:36 | 3:29 |  | | 4:07^†^ | 4:10^†^ |
| Insomnia severity index (ISI) | 3.2 | 4.2 |  | | 9.1^†^ | 9.6^†^ |
| Moderate to severe insomnia(ISI≥15), % | 2.7 | 5.3 |  | | 11.7^†^ | 13.5^†^ |
| Epworth sleepiness scale (ESS) | 5.5 | 5.6 |  | | 6.7^†^ | 6.3^†^ |
| Excessive daytime sleepiness(ESS>10) | 11.9 | 11.1 |  | | 15.1^†^ | 10.6 |

^†^Significant changes compared to the pre-pandemic period (p<0.05)
